# Degradation of cognitive timing mechanisms in behavioural variant frontotemporal dementia

**DOI:** 10.1016/j.neuropsychologia.2014.10.009

**Published:** 2014-12

**Authors:** Susie M.D. Henley, Laura E. Downey, Jennifer M. Nicholas, Kirsi M. Kinnunen, Hannah L. Golden, Aisling Buckley, Colin J. Mahoney, Sebastian J. Crutch

**Affiliations:** a Dementia Research Centre, Department of Neurodegenerative Disease, UCL Institute of Neurology, University College London, London WC1N 3BG, United Kingdom; b National Hospital for Neurology and Neurosurgery, UCLH NHS Foundation Trust, Queens Square, London WC1N 3BG, United Kingdom; c Department of Medical Statistics, London School of Hygiene and Tropical Medicine, Keppel Street, London WC1E 7HT, United Kingdom

**Keywords:** Motor timing, Finger tapping, Frontotemporal dementia, Cerebellum

## Abstract

The current study examined motor timing in frontotemporal dementia (FTD), which manifests as progressive deterioration in social, behavioural and cognitive functions. Twenty-patients fulfilling consensus clinical criteria for behavioural variant FTD (bvFTD), 11 patients fulfilling consensus clinical criteria for semantic-variant primary progressive aphasia (svPPA), four patients fulfilling criteria for nonfluent/agrammatic primary progressive aphasia (naPPA), eight patients fulfilling criteria for Alzheimer׳s disease (AD), and 31 controls were assessed on both an externally- and self-paced finger-tapping task requiring maintenance of a regular, 1500 ms beat over 50 taps. Grey and white matter correlates of deficits in motor timing were examined using voxel-based morphometry (VBM) and diffusion tensor imaging (DTI). bvFTD patients exhibited significant deficits in aspects of both externally- and self-paced tapping. Increased mean inter-response interval (faster than target tap time) in the self-paced task was associated with reduced grey matter volume in the cerebellum bilaterally, right middle temporal gyrus, and with increased axial diffusivity in the right superior longitudinal fasciculus, regions and tracts which have been suggested to be involved in a subcortical–cortical network of structures underlying timing abilities. This suggests that such structures can be affected in bvFTD, and that impaired motor timing may underlie some characteristics of the bvFTD phenotype.

## Highlights

• We examine motor timing in behavioural variant FTD.
• Patients with behavioural variant FTD showed impaired motor timing.
• Patients tended to tap faster than target and speed up during the task.
• Faster tapping was associated with reduced grey matter in the cerebellum.
• Impaired timing might underlie some other behavioural features of FTD.

## 1 Introduction

Frontotemporal dementia (FTD) represents the second most common cause of early-onset dementia after Alzheimer׳s disease, and can be phenotypically classified as being either a syndrome of primary progressive aphasia, or as a pervasive dysfunction in normal behaviour and comportment (Warren et al., 2013). Behavioural variant frontotemporal dementia (bvFTD) manifests as progressive corrosion of normal social interaction and cognitive function. The neurobiological basis for the selective degradation of neural circuits mediating the phenotypic expression of bvFTD is poorly understood. Early diagnosis is often impeded by insidious onset and progression, and phenotypic confusion with other dementia diseases or psychiatric illness (Balsis et al., 2005; Glosser et al., 1995; Gregory et al., 1999; Mesulam, 2001, 2007; Snowden et al., 2003). It is estimated that up to 40% of all cases of frontotemporal dementia (FTD) are caused by an underlying genetic mutation (Rohrer et al., 2010). The most predominant responsible genes are the microtubule associated binding protein tau (MAPT, Hutton et al., 1998), which leads to a cascade of hyperphosphorylated tau; mutations in the gene encoding progranulin (PGRN), which causes FTD with ubiquitin and TDP43 inclusions, and the recently-identified expansion of the chromosome 9 open reading frame 72, which is defined by TDP43 proteinopathy (C9ORF72, DeJesus-Hernandez et al., 2011; Majounie et al., 2012). Various neuropsychological measures have been applied to the study of bvFTD in order to identify the earliest presenting deficits and whether any specific neuropsychological measures may be used as markers of disease manifestation and progression. Extensive examination of this population suggests that the earliest and most prominent features of the disease include a degradation of social cognition and behaviour, and deficits in attention, planning, and executive function (Rascovsky et al., 2011; Snowden et al., 2003).

Precise timing is essential for many human behaviours (Bueti et al., 2008). Important mental functions involving timing include perception and encoding of temporal information, attention shifting, storage and retrieval from long-term memory, and comparison of the temporal memory with other stored templates (Allman et al., 2011; Allman and Meck, 2012; Grondin, 2010). It is also postulated to contribute to theory of mind (Baron-Cohen et al., 2001). Each of these processes are deficient in bvFTD to some extent, and thus it may be possible that such hallmark deficits observed in this heterogeneous syndrome are at least partially mediated by loss of impairments in or changes to a subjective sense of time and the ability meaningfully to perceive and monitor behaviour according to an implicit timing mechanism (Allman and Meck, 2012; Buonomano and Karmarkar, 2002). Timing has been used as a model system of cognitive dysfunction in neurological disease states, with disorders of time and perturbations in timing mechanisms observed in a number of neurological conditions including Parkinson׳s disease (O׳Boyle et al., 1996; Perbal et al., 2005), Huntington׳s disease (Hinton et al., 2007; Rowe et al., 2010), schizophrenia (Davalos et al., 2005), frontal lesion patients (Picton et al., 2007) and a single case study of a patient with frontotemporal dementia (Wiener and Coslett, 2008).

There are many different aspects of timing, for example perceiving time, predicting time, being oriented in time, as well as different scales which probably involve different mechanisms (for example sub-second and supra-second timing). Much work has been done to elucidate the neuroanatomical bases underlying human timing in both healthy individuals and those with focal and degenerative brain lesions. Recent reviews on various aspects of timing (Coull et al., 2011; Ortuño et al., 2011) highlight the role of the motor and supplementary motor areas as integral components of a larger thalamo-cortico-striatal cognitive timing circuit. Results from studies in Parkinson׳s disease (PD) (Harrington and Haaland, 1999; O׳Boyle et al., 1996) and cerebellar lesion patients (Spencer et al., 2003), as well as in healthy controls (Bueti et al., 2012; Coull et al., 2013; Gilaie-Dotan et al., 2011; Hayashi et al., 2014; Lewis and Miall, 2003) are often interpreted as reflecting the involvement of both the cerebellum and basal ganglia in broader time-keeping operations (Bueti et al., 2008), suggesting a common network of timing-related areas underpinning the use of time both for action and perception.

Coull and colleagues draw attention to the necessity of differentiating between implicit and explicit timing requirements when examining the neurological substrates of these supposedly biologically-separable concepts. The work presented here focuses on supra-second “motor” timing, a measure of explicit (overt) timing requiring participants to estimate a time interval and produce some overt response (often finger tapping). Coull et al.׳s recent review presents evidence that for this form of explicit timing the basal ganglia encode a representation of the stimulus, the supplementary motor area (SMA) is engaged by “online” timing (accumulation of stimulus duration, and hence predicting when to make the motor response) and that frontal cortex is also involved, although findings here are less consistent, and may also indicate the contribution of attention and working memory to supra-second timing tasks. The cerebellum is also posited to play a role in representing duration, particularly in short motor timing tasks where demands on attention and memory are relatively lower than for longer times (Coull et al., 2011; Ivry and Spencer, 2004), although the duration at which cortical input might become more important than subcortical is still debated (see e.g. Witt et al., 2008).

An fMRI study of a self-paced finger tapping task highlighted the role of the SMA, basal ganglia, and right-lateralised frontal and parietal cortices (Coull et al., 2013). This functional specificity of neural regions involved in timing maps onto the “pacemaker-accumulator” information processing account of timing (Gibbon et al., 1984). This is also supported by studies of self-paced tapping in patient populations in which the supplementary motor area (SMA), premotor cortex (PMC), dorsolateral prefrontal cortex (DLPFC), and basal ganglia (BG) were thought to subserve this function (Bechtel et al., 2010; Harrington & Haaland, 1999; Rowe et al., 2010), although a recent meta-analysis only found evidence for basal ganglia involvement in sub-second timing tasks (Wiener et al., 2010).

More recently, emerging evidence suggests that white matter tracts interconnecting the cortical areas implicated in cognitive timing may also play a key role in timing functions (Bueti et al., 2012), including motor timing (Schulz et al., 2014; Ullén et al., 2008). It is likely that motor timing is subserved by a network of cortical and sub-cortical structures. Damage to any one of the components of the cognitive timing circuit, including its anatomical connections, could cause dysfunction in motor timing ability.

Many of the aforementioned structures implicated in paced motor timing ability (Coull et al., 2013; Witt et al., 2008) represent a constellation of anatomical regions that are consistently targeted by FTD pathology, suggesting that paced timing tasks may be sensitive to FTD-related dysfunction. One of the earliest sites of pathological involvement in bvFTD is the striatum (Snowden et al., 2003), an area proposed as the ‘core timer’ within a cortico–thalamo–striatal cognitive timing network (Allman and Meck, 2012). Although the cerebellum is not commonly conceptualised as a centre of pathology in bvFTD, this structure has recently been implicated in the pathogenesis of the FTD-MND gene c9ORF72 (DeJesus-Hernandez et al., 2011; Mahoney et al., 2012; Majounie et al., 2012). Indeed a recent report suggests that in cases with c9ORF72 mutations, of which bvFTD is the most common phenotype, the cerebellum is one of the earliest and most prominent sites of pathological deposition and subsequent degradation (Mahoney et al., 2012).

Although bvFTD is phenotypically, pathologically and genetically highly heterogeneous, imaging studies suggest that the underlying neurodegeneration and spread of pathological deposition follows a somewhat predictable trajectory (Hornberger et al., 2012). Studies using techniques such as diffusion tensor imaging (DTI) suggest that white-matter degradation can be more extensive than grey matter atrophy in the early stages of bvFTD (Agosta et al., 2012), and that tract degradation follows a somewhat predictable atrophic trajectory (Agosta et al., 2012; Zhang et al., 2013). Grey matter structures particularly relevant to the proposed cognitive timing circuitry, including the basal ganglia and cerebellum, are spread within cerebral white matter; however, to the best of our knowledge, no group study has identified an association between cognitive timing ability and integrity of underlying white-matter tracts, in patients with bvFTD.

The current study employs finger-tapping tasks to examine one aspect of cognitive timing, “motor timing”: overt reproduction of an interval. Finger-tapping tasks require participants to button-press in time with a paced metronome (externally-paced), or to keep that beat once the metronome has ceased (self-paced), and are often used to assess timing ability. Such tasks have been shown to provide invaluable measures for tracking the manifestation and progression of disease in persons prodromal to and affected by both Huntington׳s disease (Bechtel et al., 2010; Rowe et al., 2010) and Parkinson׳s disease (O׳Boyle et al., 1996), and have been used to explore the differential effects of focal frontal lesions on timing performance (Picton et al., 2007).

Several statistical models have been proposed to evaluate performance on such tasks. The most widely-accepted approach is that offered by Wing and Kristofferson (1973), which purports that time can be parcelled out at the neural level into clock and motor contributions to timing ability and variability. Studies of patients with neurological damage have provided some evidence that the two processes of motor and clock variance can be dissociated and mapped onto discrete neural systems (Ivry and Spencer, 2004; Spencer et al., 2003). Continued investigation of timing in neurological populations may further elucidate both neurological timing mechanisms and provide novel metrics of understanding disease manifestation and progression.

The present study aimed to assess the ability of a cohort of patients with bvFTD to keep time under externally-paced and self-paced conditions relative to healthy controls, and also compared their performance with patients with Alzheimer׳s disease (AD) and the language variants of FTD; semantic variant primary progressive aphasia (svPPA) and nonfluent/agrammatic primary progrssive aphasia (naPPA). Grey and white matter correlates of timing ability in bvFTD were assessed using voxel-based morphometry (VBM) and diffusion tensor imaging (DTI) respectively. It was hypothesised that in comparison to both healthy individuals, and a population of neurologically-compromised disease controls, bvFTD patients would be able to keep the beat with an externally-paced tone, but would show a deficiency in their ability to maintain a regular supra-second beat without the aid of external cues in a self-paced task. Should such a dysfunction be observed, it was hypothesised to emanate from a disruption of the underlying cortico–subcortical circuit subserving timing mechanisms, including both grey and white matter, which we propose to be degraded in bvFTD. We therefore assessed the relationship between tapping performance and grey matter structure across the whole brain, in order to investigate any neural correlates of tapping in an unbiased way. We subsequently (without reference to the whole-brain findings) examined these relationships within the supplementary motor area (SMA). Of the key areas purported to be involved in supra-second motor timing (the basal ganglia, SMA and right fronto-parietal regions) the SMA is most consistently implicated and hence was the area on which we focused (Coull et al., 2013; Ortuño et al., 2011; Wiener et al., 2010). We also investigated the structure of white-matter tracts connecting the cortical and subcortical components of the proposed cognitive timing network, consisting of the cerebellum, basal ganglia, motor and premotor areas and the prefrontal cortex.

## 2 Methods

### 2.1 Participants

Twenty patients fulfilling consensus criteria for bvFTD (Rascovsky et al., 2011) and with structural MRI evidence of atrophy in support of their syndromic diagnosis were recruited to the study. In addition, 11 participants fulfilling consensus criteria for semantic variant primary progressive aphasia (svPPA, Gorno-Tempini et al., 2011), four participants fulfilling consensus criteria for nonfluent/agrammatic primary progrsesive aphasia (naPPA, Gorno-Tempini et al., 2011), and eight participants fulfilling consensus criteria for Alzheimer׳s disease (AD, Dubois et al., 2014) were recruited as disease controls. All patients were recruited from the tertiary-level Specialist Cognitive Disorders Clinic at the National Hospital for Neurology and Neurosurgery, London, United Kingdom (demographic details summarised in Table 1). Thirty-one healthy control participants with no history of neurological or psychiatric illness were also recruited. Informed consent was obtained for all participants and the study was approved by the local research ethics committee under Declaration of Helsinki guidelines.

### 2.2 Behavioural assessment

#### 2.2.1 Standard neuropsychological assessment

All participants had an assessment of general neuropsychological function, consisting of the following standard clinical neuropsychological tests: National Adult Reading Test (NART, Nelson, 1982); Wechsler Abbreviated Scale of Intelligence (WASI, Wechsler, 1999); Recognition Memory Test (RMT, Warrington, 1984); digit span forwards and backwards (from the WAIS-R, Wechsler, 1981); British Picture Vocabulary Scale (BPVS, Dunn et al., 1997); Graded Naming Test (GNT, McKenna and Warrington, 1983); Graded Difficulty Arithmetic (GAD, Jackson and Warrington, 1986); the object decision subtest of the Visual Object and Space Perception Battery (VOSP, Warrington and James, 1991); the Stroop colour-reading, word-reading and interference conditions from the Delis–Kaplan Executive Function System (D–KEFS, Delis et al., 2001).

On rare occasions some patient participants were unable to complete some of the above tests owing to the nature of their impairment and corresponding numbers are reported in Section 3. Only participants who performed above floor on the majority of background tests, including tests of comprehension, were administered the tapping task in order to ensure that the tapping task was adequately understood.

#### 2.2.2 Experimental procedure

The experimental test was administered under Superlab© on a Dell OptiPlex 960 computer. Tone stimuli were presented in free field at a comfortable listening level for each participant (at least 70 dB). The timing of finger taps was recorded using a Cedrus^®^ RB-730 Response Pad. Conditions were presented in fixed order (externally-paced followed by self-paced). Participant responses were recorded and stored for offline analysis. A single repeat of task was allowed if the examiner considered that the participant had been distracted during the original presentation or did not adequately understand the task requirements. No feedback about performance was given during the session.

The majority of participants did the tapping task on the same day as the standard neuropsychological assessment (median time between assessments=0 days). Eight controls and one bvFTD patient did the tapping task more than 3 months after the background assessment (time between assessments ranged from 99 to 244 days for the eight controls, and was 129 days for the bvFTD patient).

#### 2.2.3 Externally-paced tapping task

Participants were informed that they would hear a series of tones, and that these would be played according to a regular beat [1500 ms intervals; 0.67 Hz]. Participants were instructed to use the index finger of their dominant hand to tap in pace with these tones, and further, that because the tones were produced according to a regular beat, that they should start to be able to predict when each tone was to be played and thus tap at the same time as the production of the tone. Participants were given a practice session in which a series of six auditory stimuli separated by the fixed interval of 1500 ms was presented, and subsequently presented with a total of 50 auditory stimuli.

#### 2.2.4 Self-paced tapping task

After completion of the externally-paced tapping task, participants were instructed that they would be presented with a short succession of tones, with which they should similarly keep pace using the index finger of the dominant hand. Participants were informed that these tones would cease after a short period and that they would be required to keep the beat going by continuing to tap at the same pace until they were instructed to stop. The synchronization phase consisted of six auditory stimuli separated by a fixed interval of 1500 ms, following which participants were required to continue tapping at the established pace for a further 50 taps.

### 2.3 Analysis of behavioural data

Behavioural data were analysed using STATA release 12.0 or later (StataCorp, 2011).

#### 2.3.1 Demographic and background data

Group differences in age and NART (an index of estimated premorbid IQ) were investigated using *t*-tests with Satterthwaite׳s approximation for unequal variance. Fisher׳s exact test was used to examine whether gender differed between groups. Linear regression models with robust standard errors (to allow for difference in variance between groups) were used to compare group differences in standard neuropsychological tests, adjusting for age and gender by including them as covariates. For each test we examined pairwise differences between controls and the four patient groups, and between the bvFTD group and the three patient “control” groups, but not between any of the other patient groups.

#### 2.3.2 Tapping task

The tapping data were analysed according to the Wing and Kristofferson (1973) model of motor response timing. This model proposes that each inter-response interval, *I*, is determined by two processes: a central timekeeper or clock that provides the trigger to initiate the response at intervals *C*, and a motor system delay, *D*, between the clock trigger and the response. It is assumed that both clock interval and motor delay vary between responses and that these two processes are independent. A further assumption is that each inter-response interval is determined by only the clock interval *C* for that response and the motor delay *D* both for that response and for the preceding response, so for the *j*th response

$$I_{j}=C_{j}-D_{j-1}+D_{j}$$

The dependence of the inter-response interval on the motor delay of the previous response imposes a negative correlation between successive inter-response intervals: a short inter-response interval is expected to follow a long inter-response interval, or a long interval is expected follow a short interval. This negative lag one autocorrelation, *ρ*(1), allows separation of the clock and motor delay components of the timing process. It is defined as follows:

$$\rho \left(1\right)=\frac{G\left(1\right)}{G\left(0\right)}$$

where *G*(1) is the lag 1 covariance and *G*(0) is the lag 0 covariance (i.e. equivalent to variance of the inter-response interval). It is assumed that for lag greater than 1 the autocorrelations are zero, since there should be no dependence between responses separated by more than 1 interval. Based on this assumption, the variance in the motor delay and clock process can then be estimated as follows:

$$\sigma_{D}^{2}=\;-G\left(1\right)$$

$$\sigma_{C}^{2}=G\left(0\right)+2G\left(1\right)$$

If there is no motor delay *ρ*(1) will be 0, since the lag 1 covariance, *G*(1), will equal zero. If there is no clock variability *ρ*(1) will be −0.5 (half the variability determined by the motor delay of the *j*−1^th^ response and half by the motor delay of the *j*^th^ response).

Data were analysed following a modification of the Wing and Kristofferson (1973) model, to allow for change over time (drift) in the inter-response interval and for missing values. An autoregressive-moving-average model with exogenous inputs (ARMAX model) was fitted to model inter-response interval for each task for each participant, with lag 1 autocorrelation. In the paced task linear and quadratic terms for stimulus were fitted to allow for drift in inter-response interval over the task. To allow for relatively complex drifts in the self-paced tapping task, linear, quadratic and cubic terms were fitted for stimulus number. Any inter-response intervals that were more than two standard deviations from the predicted value of the model were excluded, since these were thought to be due to error in data collection (missed taps or accidental double taps). For all analyses we removed the first two taps in the externally-paced and the first seven taps in the self-paced task in order to eliminate atypical early responses.

For each participant for each task, we assessed the following performance measures: inter-response interval (IRI), inter-response interval variance, clock variance, motor variance, response interval drift and response interval absolute drift. IRI was assessed through the mean modelled inter-response interval. Variance in inter-response interval *G*(0) was estimated by the ARMAX model residual variance, after allowing for drift. Motor and clock variance were derived from the ARMAX model *G*(1) and *G*(0) as detailed above. Drift was estimated as the difference in the modelled IRI between first and final stimulus, and can take positive (getting slower) or negative (getting faster) values. Absolute drift was also assessed as this gives a measure of the magnitude of drift, at the loss of information on direction.

Separation of motor and clock variance was considered to have limited validity for the self-paced task, as descriptive analysis suggested that the motor component was minimal (mean *ρ*(1) was close to zero in all participant groups). Therefore, we compared total variance between participant groups in the self-paced task, as suggested by Ivry and Keele (1989) for situations when there is little motor element. We also provided estimates with separation into clock and motor variance to allow examination of whether our use of total variance had any impact on our findings.

Linear regression was used to model differences in task performance between participant groups. Further exploratory analysis used linear regression to examine whether there was an interaction between direction of drift (negative versus positive) and participant group in the model for absolute drift. This interaction was examined for controls and participants with bvFTD only since the other participant groups were very small when separated into different drift direction strata. Models were fitted both with and without adjustment for gender and age. Due to the non-Normal distribution of performance measures, non-parametric, bias-corrected and accelerated bootstrap confidence intervals were used, calculated from 2000 replications.

#### 2.3.3 Associations between cognitive skills and tapping measures

In order to investigate whether tapping performance was related to other cognitive skills linear regression models were used to assess the relationship between tapping measures and Stroop Interference score (an index of executive function) and Digit Span Backwards (an index of working memory). The tapping measures of interest were mean IRI for both externally-paced and self-paced tapping; clock variance (externally-paced tapping) and total variance (self-paced tapping, because motor variance was negligible in this task and could not be separated from clock variance); and drift and absolute drift (both tasks). Note that the drift metric can be both positive (getting slower through the task) and negative (getting faster through the task). Therefore, group average drift may be close to zero, even in the presence of substantial individual drifts, if participants are equally likely to drift in the positive or negative direction, hence for between-group comparisons we used absolute drift. However, associations between drift and other variables of interest are of more relevance because we can assess whether getting slower or faster is associated with better or worse scores. Absolute drift, on the other hand, indicates the amount of change both in participants who get faster over time, and those who get slower, but loses direction information, and therefore allows us to assess differences in group mean magnitude of drift and also whether “losing time” (in any direction) is associated with other measures.

Models were fitted to assess the linear relationship between tapping measures and score in the control group, the bvFTD group and their interaction, i.e. assessing whether this relationship differed between the two groups. As with other analyses age and gender were included as covariates. Again because of the non-Normal distribution of tapping measures non-parametric, bias-corrected and accelerated bootstrap confidence intervals were estimated, calculated from 2000 replications.

### 2.4 MRI acquisition

Brain MRI data were acquired for 19 of the bvFTD participants on a Siemens Trio 3T MRI scanner using a 32-channel phased array head-coil (Siemens, Erlangen, Germany). A sagittal 3D magnetisation prepared rapid gradient echo T1 weighted volumetric MRI (TE/TR/TI=2.9/2200/900 ms, dimensions of 256×256×208, voxel size of 1.1×1.1×1.1 mm^3^) and a coronal fluid attenuated inversion recovery (FLAIR) sequence were acquired. In addition, two 64-direction diffusion-weighted imaging (DWI) sequences were acquired with a single shot, spin-echo echo planar imaging sequence (FOV: 240 mm; matrix: 96×96; yielding an isotropic voxel size of 2.5×2.5×2.5 mm^3^; 55 contiguous axial slices; TR: 6800 ms; TE: 91 ms; *b*-value: 1000 s/mm^2^; 9 images with *b*=0 s/mm^2^), augmented with parallel imaging acceleration (GRAPPA) to reduce susceptibility artefact. For all patient participants, volumetric MRI, FLAIR and DWI sequences were assessed visually in all planes to ensure adequate coverage and to exclude artefacts, unexpected pathology or significant motion. Imaging data were not acquired for control participants.

Two patient images were not analysed because of bad movement artefact. Of the 17 remaining images the majority were acquired within a week of the tapping task being administered (median time between scan acquisition and tapping=7 days). All patients underwent scanning within 1 month of doing the tapping task, with the exception of one patient who was scanned just over 4 months prior to doing the tapping task. One participant in the bvFTD group who had a scan was unable to complete the self-paced tapping task; therefore the self-paced imaging analyses included only 16 images, whilst the externally-paced analyses included 17.

### 2.5 Imaging analysis

Imaging data were analysed in the bvFTD participants only. This was partly because this was the main group of interest, and also because small numbers in the AD and naPPA groups in particular precluded imaging analysis.

Image analyses were conducted to examine only correlations between brain volume (VBM) or white matter structure (DTI) and those behavioural metrics where bvFTD participants performed significantly worse than controls, which were clock variance and absolute drift on the externally-paced task, and mean IRI and absolute drift on the self-paced tasks.

#### 2.5.1 Voxel based morphometry (VBM) image analysis

##### 2.5.1.1 Image pre-processing

Pre-processing of brain MR images was performed using the DARTEL toolbox within SPM12 (www.fil.ion.ucl.ac.uk/spm) running under MATLAB R2012a (www.matlab.com). Using the “Segment” routine in SPM12, native-space whole-brain MR images were segmented into native-space grey matter (GM), white matter (WM) and CSF, and rigidly-aligned GM and WM segments. Bias-corrected whole brains in native space were also output. The rigidly-aligned GM and WM segments were used to create DARTEL templates using the “run DARTEL (create Templates)” command under DARTEL tools. Finally the “Normalise to MNI space” command was used with 1 mm isotropic voxel size to warp into MNI space, modulate and smooth (6 mm full-width half-maximum) the GM segments prior to statistical analysis.

The bias-corrected whole brains in native space were also warped into MNI space (with 1 mm isotropic voxel size) using “Normalise to MNI space” and then averaged in order to create a study-specific template for displaying results. To help protect against voxel drop-out because of potentially marked local regional atrophy in particular scans, a customised explicit brain mask was derived by maximising the correlation between the binary mask and the average of the images to be analysed (Ridgway et al., 2009), using the “masking” toolbox in SPM12. This mask was applied to the images prior to statistical analysis as part of the “Randomise” command from the FMRIB Software Library (FSL v5.0.7, Jenkinson et al., 2012) (see below).

The “tissue volumes” routine in SPM12 was used to calculate volumes of GM, WM and CSF from the initial segmentation files, and these were summed to provide a measure of total intracranial volume (TIV). This was used as a covariate in order to adjust for differences in head size in subsequent analyses (see below).

An anatomical small volume of the supplementary motor area (SMA) was derived in FSL by manually outlining the SMA from the Harvard–Oxford cortical atlas tool (Desikan et al., 2006) and saving the resulting region as a binary image. The volume of this region was 11,949 voxels (11,949 mm^3^).

Parameter estimation and statistical testing for each of the models described in Section 2.5.1.2 (below) were first done across the whole brain and then (independently of whole-brain findings) within the SMA region of interest, by masking whole-brain images with the SMA region prior to analysis.

##### 2.5.1.2 Statistical analysis

Linear regression models were used to examine the association between regional grey matter volume and the four tapping scores outlined above (see section 2.5) in the bvFTD group. Voxel intensity (*V*, an index of grey matter volume) was modelled as a function of tapping score, adjusting for the effects of participant age, gender and TIV by including them as covariates.

$$V=\;\beta_{1\;}\mathrm{score}+\;\beta_{2}\mathrm{age}+\;\beta_{3}\mathrm{gender}+\;\beta_{4}\mathrm{TIV}+\;\mu +\;\varepsilon$$

where *μ* represents a constant and *ε* the error term. Separate models were used to assess grey matter associations for each of the scores of interest (i.e. four separate models examining: externally-paced clock variance; externally-paced absolute drift; self-paced mean IRI; self-paced absolute drift).

In each model the contrast (statistical test) of interest was the one-tailed *t*-test comparing the parameter estimating the relationship between the experimental score and grey matter volume (*β*_1_) against zero. We predicted that higher absolute drift (indicative of worse performance) would be correlated with lower brain volume, i.e. we looked for a negative correlation between absolute drift and brain volume. Similarly we predicted that higher clock variance (indicative of worse performance) would be correlated with lower brain volume, i.e. we looked for a negative correlation between these two variables. In the model above this is the contrast *β*_1_<0. Most bvFTD patients tended to show faster-than-target self-paced tapping (faster mean IRI) and therefore we predicted that faster (lower) mean IRI (farther from the 1500 ms target) would be associated with reduced grey matter volume (the contrast *β*_1_>0). As is good practice, we also investigated the “reverse” contrasts, i.e., looked for evidence of correlations in the non-predicted direction for each of these variables.

All analysis (statistical and thresholding) of VBM data was implemented in FSL. Contrasts were tested using the permutation-based (nonparametric) Randomise (v2.9) tool within FSL (Winkler et al., 2014) with 5000 permutations generated for each test and variance smoothing with a standard deviation of 3 mm. This was used because this method does not assume Normality of data or stationary smoothness (which is often the case for imaging data), and is still valid with relatively small numbers.

Results were adjusted for multiple comparisons using family-wise error (FWE) correction with threshold-free cluster enhancement in FSL (Smith and Nichols, 2009), thresholded at *p*<0.05. This method of correction for multiple comparisons was used both for the whole-brain analysis and the subsequent small volume analyses.

#### 2.5.2 Diffusion tensor (DTI) image analysis

##### 2.5.2.1 Image pre-processing

Diffusion image pre-processing, including image co-registration and spatial normalisation, was performed using tools from FSL. First, the diffusion-weighted images from the two sequences were corrected for eddy currents and motion by registering these to the same non-diffusion-weighted (*b*=0) reference image using FLIRT (Jenkinson and Smith, 2001; Jenkinson et al., 2002) with spline interpolation and all angular search ranges set to 0. Each participant׳s reference *b*=0 image and T1-weighted structural image were then co-registered using FLIRT with default settings. As the patients׳ brains were affected by atrophy, a template brain mask (MNI152) was used for deriving masks for the subsequent processing of the patients׳ diffusion images. FLIRT was first used to register by affine transformation a patient׳s skull-stripped T1-weighted image to the template MNI152 T1 image in FSL, and the resulting transformation input into FNIRT, used to nonlinearly warp the native-space image into the standard space. The two transformations (from the reference *b*=0 to the T1 and from the native to the standard T1) were next combined, and this transformation inverted and finally applied to bring the MNI152 brain mask into the patient׳s native diffusion space. The diffusion tensor model was fitted on the data using a weighted linear approach, and three-dimensional fractional anisotropy (FA), mean diffusivity (MD), axial diffusivity (AX) and radial diffusivity (RD) images derived from the tensor eigenvalues *λ*1, *λ*2 and *λ*3.

##### 2.5.2.2 Tract-based spatial statistics

Tract-based spatial statistics (TBSS; (Smith et al., 2006) was used for voxel-wise analysis of white matter tract structure. The patients׳ FA images were nonlinearly aligned into FSL׳s FMRIB58 template space, and the aligned images affine-transformed into MNI152 space. The patients׳ FA data were then projected onto the FMRIB58 mean FA ‘skeleton’, representing the brain׳s major white matter tracts (thresholded at FA≥0.2), to derive an ‘all_FA_skeletonised’ image. This four-dimensional image, containing data from all patients, was used for the voxel-wise statistical analysis. Skeletonised data images were similarly obtained from the patient׳s mean, axial and radial diffusivity images. To restrict the analysis to only the a priori defined cognitive timing network, a ‘cognitive timing mask’ was built using fslmaths, from the JHU ICBM-DTI-81 white matter labels atlas (Mori et al., 2008) in FSL. This consisted of tracts connecting the most commonly-implicated structures within a generously-defined network (Coull et al., 2011; Ortuño et al., 2011): the cerebellum, basal ganglia, motor and premotor areas, and the prefrontal cortex. The tracts were the left and right cerebral peduncle, inferior and superior cerebellar peduncles, superior longitudinal fasciculus (SLF), superior fronto-occipital fasciculus (SFO), uncinate fasciculus (UF), and the cingulum bundles. Each of the tracts was selected from the atlas, thresholded and saved in the MNI152 standard space. Each tract was then binarised for adding them together to derive a cognitive timing mask that contained all tracts of interest. This mask was intersected with a binary mask of the thresholded mean FA skeleton, and then used in the statistical analyses of correlations between the DTI measures and behavioural scores.

##### 2.5.2.3 Statistical analysis

Similarly to the VBM analysis (Section 2.5.1), a general linear model was used with tapping score as the factor of interest and age and gender as nuisance covariates. Separate models were used for each of the four tapping scores, and this model was fitted separately to FA, MD, AX, and RD data: thus 16 models were fitted. As for the VBM analysis described above, non-parametric permutation-based statistics were employed using Randomise in FSL with variance smoothing (standard deviation of 1 mm) and 5000 random permutations generated for each test. Results were adjusted for multiple comparisons using threshold-free cluster enhancement (Smith and Nichols, 2009), and a threshold of *p*<0.05 was used.

## 3 Results

### 3.1 Demographic characteristics

Demographic and task-related information is presented in Table 1. There was no evidence that mean age differed between controls and the four patient groups, or between the bvFTD group and the other patient groups (all *p*>0.099). Proportions of males and females differed between groups (*p*=0.022) most likely reflecting the fact that females were under-represented in most patient groups compared with the control group. On average, all patient groups showed worse performance at the NART than the control group (all *p*<0.013); for this reason NART (often used as an index of premorbid IQ) was not used as a covariate in any further analysis.

### 3.2 General neuropsychological performance

Neuropsychological performance is presented in Table 1. In general all patient groups were impaired, on average, compared with healthy controls on all tests (*p*<0.05 all comparisons), after adjusting for age and gender. Exceptions were that the naPPA group was not impaired relative to controls at RMT Words (*p*=0.32), RMT Faces (*p*=0.075), and VOSP object decision (*p*=0.28), nor at GNT or BPVS although there were trends towards this (GNT: *p*=0.052; BPVS: *p*=0.055). Thus all patient groups were impaired across most domains, with some sparing of memory, naming, comprehension and visuo-perceptual skills in the naPPA group.

Comparison of the bvFTD group with other patient groups was suggestive of disproportionate deficits in the domains particularly affected by each syndrome: the svPPA group was worse than the bvFTD group at tests tapping semantic knowledge (WASI Vocabulary, Similarities, BPVS, and GNT, all *p*<0.05); the naPPA group was worse than the bvFTD group at tests requiring time-limited verbal output (GDA both addition and subtraction, Stroop colour and word although not inhibition) and better than the bvFTD group at tests of memory, comprehension, naming and visuo-perceptual skills (RMT words, RMT faces, BPVS, GNT, and VOSP); and the AD group was worse than the bvFTD group at Stroop inhibition (note this is time-to-complete, not errors), and digit span backwards, although slightly better at confrontation naming (GNT) (all *p*<0.05).

### 3.3 Tapping task

A number of participants completed only the externally-paced component of the task due to an inability to sustain attention on the self-paced task without the guidance of the tone stimuli. Three controls, 1 bvFTD and 2 svPPA participants were unable to complete the self-paced task.

#### 3.3.1 Externally-paced tapping

**Fig. 1:**
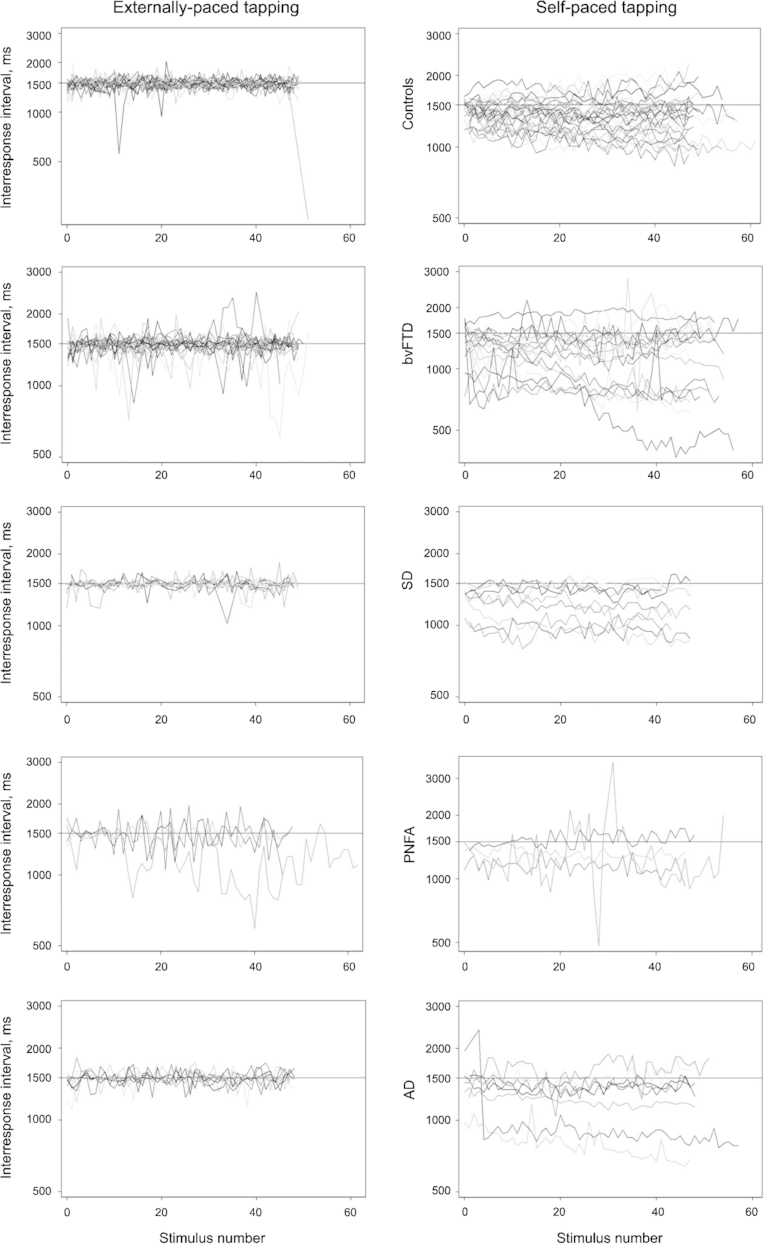
Plots of externally-paced (left panels) and self-paced (right panels) unadjusted interresponse interval for both tapping tasks, each row showing controls, bvFTD, svPPA, naPPA and AD respectively.

Fig. 1 shows the inter-response intervals by stimulus for each participant in each group. Table 2 gives the summary statistics for each timing metric. In all groups most participants׳ IRIs during externally-paced tapping started close to the fixed interval of 1500 ms and showed little drift over the task. The lag 1 autocorrelation for the majority of participants was within the range from −0.5 to 0, as would be expected from theoretical assumptions of the Wing and Kristofferson model. The negative minimum clock and motor variance reported in Table 2 results from the minority of participants with lag 1 autocorrelation outside of this range.

Mean differences between controls and patient groups for the tapping measures are shown in Table 3. Compared with controls, mean clock variance was higher for participants with bvFTD (mean difference 13,039 ms^2^; 95% CI 3028, 26,643 ms^2^ after adjusting for age and gender) but not significantly different in any of the other patient groups. Motor variance did not differ significantly from controls in all patient groups. There was no evidence that mean IRI differed between controls and patient groups, although there was a trend towards the svPPA group being very slightly slower than controls (mean difference 6 ms; 95% CI 0, 19 ms after adjusting for age and gender).

There were no statistically significant differences in mean drift between the controls and the patient groups (but note that “average” drift has limited relevance for group mean comparisons since it had both negative and positive values). However, patients with bvFTD showed greater absolute drift, indicating a significantly higher absolute difference between the first and last response (regardless of whether they got slower or faster), in comparison to controls, although the difference was relatively small (mean adjusted difference 38 ms; 95% CI 5, 99 ms). There was no evidence to indicate a difference between other patient groups and controls in mean absolute drift.

When assessing positive and negative drift separately participants with bvFTD had greater positive drift (mean adjusted difference 34; 95% CI 5, 93 ms) and negative drift (mean adjusted difference 61; 95% CI 8, 194 ms) than controls. There was no evidence of an interaction (i.e. there was no evidence that the amount of difference in positive drift between these two groups differed from the amount of difference in negative drift; interaction 27, 95% CI −28, 153 ms).

In summary, in the externally-paced tapping task, there was no evidence that any patient groups differed from controls with the exception of the bvFTD group which, on average, showed greater clock variance and greater absolute drift (after adjusting for age and gender). Most participants got slightly slower over time, and the magnitude of this slowing down was greater in the bvFTD group than in controls. Some participants got faster over time, and again the magnitude of this speeding up was greater in the bvFTD group than in controls.

#### 3.3.2 Self-paced tapping

Fig. 1 shows the inter-response intervals by stimulus for each group and Table 4 provides summary statistics from the autocorrelation model. For the self-paced task the assumptions of the Wing and Kristoffferson model were violated for several controls and patients, as they had *ρ*(1) greater than 0, but the proportions with violations did not differ between participant groups (*p*=0.28, Fisher׳s exact test). Most participants׳ IRIs at the start of self-paced tapping were relatively close to the fixed interval of 1500 ms. However, there were more participants in the patient groups with IRIs noticeably shorter than 1500 ms at the start of the task. A large number of participants in all groups showed drift during the task, with more participants showing negative drift (getting faster) than positive drift (getting slower). The average lag 1 autocorrelation was close to zero, with many participants having positive lag 1 autocorrelation resulting in negative estimates for motor variance. The average motor variance was close to zero in most patient groups, which suggests that in the self-paced task motor variance had little impact on the overall variance of the inter-response intervals and hence the analysis focuses on total variance. However, both these measures are reported (as well as total variance) so that they can be compared directly with the externally-paced tapping results. Between-group differences in these measures are shown in Table 5.

After adjusting for age and gender there was no evidence of any differences between patient groups and controls for any of the variance measures for this task, with the exception of the naPPA group which showed greater total variance (mean difference 48,580 ms^2^; 95% CI 292, 195,303 ms^2^); however the very small *N* and wide confidence intervals mean that this estimate should be treated with some caution.

The bvFTD group had, on average, faster mean IRIs than the control group after adjusting for age and gender (mean difference −175 ms; 95% CI −360, −26 ms); all patient groups tended to be faster than controls (i.e. to tap before the target 1500 ms interval) but the bvFTD group were the fastest and the only group for which this difference was statistically significant.

There were no significant differences between any of the patient groups and controls in drift. On average, participants with bvFTD showed greater absolute drift after adjusting for age and gender, indicating a greater absolute difference between their first and last response, in comparison to controls, with an adjusted average of 168 ms greater drift by the end of the task (95% CI 38, 322 ms). The AD group also showed greater absolute drift than controls, of a similar magnitude (mean difference 160 ms; 95% CI 14, 580 ms), but there was no evidence of this in the svPPA or naPPA groups.

Among the participants with negative drift, there was greater drift for bvFTD than controls (mean adjusted difference 225; 95% CI 91, 419 ms) but among those with positive drift this trend did not reach statistical significance (mean adjusted difference 131; 95% CI −73, 522). There was no evidence that the difference between these two group differences was statistically significant (interaction 94, 95% CI −247, 337 ms).

In summary, on the self-paced tapping task the bvFTD group had faster mean IRIs and greater absolute drift than controls; the AD group also had greater absolute drift than controls, and the naPPA group had greater total variance although this should be interpreted cautiously. The majority of participants tended to get faster on this task, and the amount of speeding up in the bvFTD participants was greater than that in controls, whilst the amount of slowing down (in the minority who slowed down) was no different to that seen in controls.

### 3.4 Associations between cognitive skills and tapping measures

For these analyses the associations between tapping measures and two cognitive tasks, Stroop interference and digit span backwards, were assessed just in the control and bvFTD groups.

#### 3.4.1 Externally-paced tapping

There was no evidence that the relationship between Stroop interference and clock variance differed between the two groups (*p*>0.05) but across both groups (controls and bvFTD combined) worse Stroop score was associated with higher clock variance (clock variance increased by 94.2 ms^2^ for each second increase in Stroop interference time, 95% CI 22.7, 290.3 ms).

The relationship between Stroop interference and drift differed between the control group and bvFTD groups (interaction, −1.7; 95% CI −3.1, −0.1) with worse Stroop performance associated with negative (faster) drift in the bvFTD group (slope −0.8; 95% CI −1.2, −0.2) but not in controls (slope 0.9; 95% CI −0.6, 2.2).

There was no evidence that mean IRI or absolute drift in this task was associated with Stroop interference score, in the bvFTD group or control group (all *p*>0.05). There was no evidence that backwards digit span was associated with performance on externally-paced tapping (accuracy, clock variance, drift and absolute drift all *p*>0.05).

#### 3.4.2 Self-paced tapping

There was no evidence that performance on the Stroop inhibition task was associated with any self-paced tapping measures (accuracy, total variance, drift and absolute drift all *p*>0.05).

Longer digit span backwards was associated with slower mean IRI across both the control and bvFTD groups (39.3 ms  increase in mean IRI for a one point increase in digit span backwards score, 95% CI 6.5, 74.4); there was no evidence that this relationship differed between the groups, *p*>0.05). There was no evidence of an association between digit span backwards and total variance, drift or absolute drift (all *p*>0.05).

### 3.5 Neuroanatomical associations

#### 3.5.1 VBM

##### 3.5.1.1 Externally-paced tapping

In the externally-paced task there were no statistically significant associations between lower grey matter volume and either increased clock variance or absolute drift, after correction for multiple comparisons across the whole brain (FWE *p*<0.05). There were also no associations in the non-predicted directions (lower grey matter volume associated with decreased variance or absolute drift). There were no statistically significant associations between either of these tapping measures and grey matter volume after small volume correction in the SMA.

##### 3.5.1.2 Self-paced tapping

**Fig. 2:**
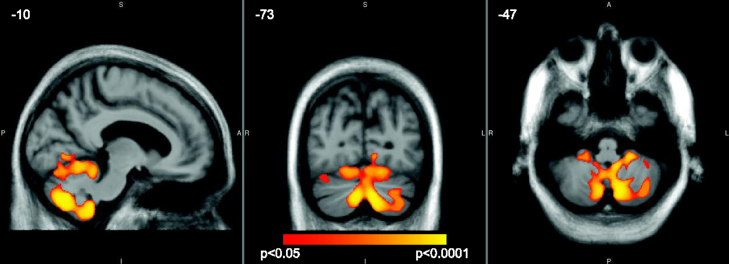
Regions in the bilateral cerebellum in which faster mean IRI in the self-paced tapping task was associated with reduced grey matter volume, *p*<0.05 (FWE correction across the whole brain). Findings are overlaid on an average image in MNI space, with coordinates in mm. The colour bar represents the corrected *p* value.

In the self-paced task there was evidence that reduced GM volume in the cerebellum and right middle temporal gyrus was associated with faster mean IRI after correction for multiple comparisons across the whole brain (FWE *p*<0.05). Peak coordinates and statistics are shown in Table 6, and the statistical parametric map in Fig. 2.

There were no statistically significant associations between reduced grey matter volume and higher self-paced absolute drift after correction for multiple comparisons. There were also no associations for either task in the non-predicted direction (i.e. no evidence that lower GM volume anywhere was associated with slower mean IRI, or that lower GM volume was associated with less absolute drift). There were no statistically significant associations between either of these tapping measures and grey matter volume after small volume correction in the SMA.

#### 3.5.2 DTI findings

##### 3.5.2.1 Externally-paced tapping

For the externally-paced tapping task there was no evidence of an association between any of the four white-matter tract measures (FA, MD, AX and RD) and either clock variance or absolute drift, after correction for multiple comparisons.

##### 3.5.2.2 Self-paced tapping

**Fig. 3:**
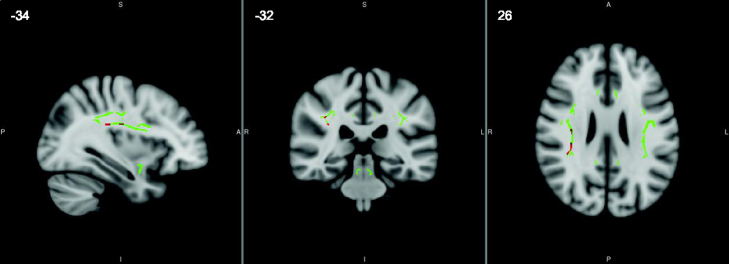
Regions in the right superior longitudinal fasciculus in which faster mean IRI in the self-paced tapping task was associated with higher axial diffusivity, after FWE correction at *p*<0.05 in the white matter tracts of interest. The tracts investigated are shown in green, and significant findings overlaid in red, on a standard 1mm voxel MNI152 brain template from FSL, with coordinates in mm.

For the self-paced tapping task faster mean IRI was associated with increased axial diffusivity in the right superior longitudinal fasciculus (*p*=0.008 at the peak voxel after correction for multiple comparisons across the tracts of interest, see Fig. 3). For the same task there was a trend towards faster mean IRI being associated with reduced fractional anisotropy in the left inferior cerebellar peduncle (*p*=0.059 at the peak voxel after correction for multiple comparisons).

There was no evidence of associations between mean IRI and other white-matter measures (MD and RD), nor any evidence of associations between absolute drift and any of the four white matter measures in this task.

## 4 Discussion

We present evidence that explicit motor timing is disrupted in bvFTD, and that this timing dysfunction is associated with degradation of some of the subcortical grey matter structures and interconnecting white matter tracts previously implicated in a cognitive timing network. As far as we are aware, this is the first cohort study to demonstrate cognitive timing dysfunction in individuals with bvFTD, and to demonstrate that this is associated with cerebellar volume, and white matter tract structure.

The present behavioural findings will first be discussed in terms of their relevance to the existing behavioural literature, and the neuroimaging findings will subsequently be considered in terms of the implications to proposed models of cognitive timing networks. Timing data were analysed according to the Wing and Kristofferson timing model (Wing and Kristofferson, 1973). Using this model we found selective impairments in aspects of externally-paced tapping in the bvFTD group; their average inter-response interval was very close to the target 1500 ms, and no different to controls, but the ‘clock’ component of their variance around the target time was much larger than that seen in controls. This group also showed more absolute drift over the task, albeit of small magnitude, suggesting that their inter-response interval changed steadily (getting either shorter or longer) over the course of the experiment, to a greater extent than that seen in controls. Thus although on average (over all the taps) the bvFTD patients anticipated the time correctly, they were not able to do so as consistently as the control group, and these deficits were not seen in the neurologically-compromised svPPA, naPPA and AD patient groups. The tendency for patients to get faster on the externally-paced task was associated with worse performance on the Stroop interference task, suggesting that an inability to inhibit an unwanted response might play a role in both impairments. For externally-paced tapping this might suggest that the primary impairment in the bvFTD group was one of response inhibition, rather than a fundamental impairment in timing ability per se, particularly given that their mean accuracy across the task was close to the target time, and not different to controls.

Self-paced timing was similarly assessed using the Wing and Kristoffersen model and aspects of this were also found to be impaired in those with bvFTD. In order for the assumptions of this model to apply, there must be some contribution above zero for both clock and motor components. On average there was little evidence for a motor variance component, so total variance was taken as an index of total clock variance. For this task total variance did not differ between controls and the bvFTD group or other patient groups (with the exception of the naPPA group although this is a very small group and confidence intervals were wide). However on this task the bvFTD group showed impaired accuracy: whilst all groups tapped, on average, faster than the target 1500 ms when there was no external stimulus, the bvFTD group was the only patient group to be significantly faster than controls. Both the bvFTD group and the AD group showed greater absolute drift on this task, and in the majority of bvFTD patients who sped up on this task (negative drift) they did so more than controls did.

Thus without external cues the bvFTD patients tend to both be too early with their response and speed up over the course of the task. In this task shorter digit span backwards was associated with faster mean IRIs (faster than the target 1500 ms) in both patients and controls suggesting that limitations in working memory capacity might contribute to faster-than-target taps, but that if so this is not a mechanism specific to bvFTD and thus is unlikely to explain fully why this group was, on average, tapping faster than controls. This finding is consistent with the assumptions of the “pacemaker-accumulator” model of timing, which suggests that working memory is used to compare an accumulated tally of pulses with a previously stored tally before a decision is made about whether the target amount of time (in an explicit motor task for example) has elapsed (Gibbon et al., 1984). However whilst this could explain worse accuracy, this does not explain the speeding up during the course of the task. Unlike in the externally-paced task, self-paced drift was not associated with performance on the Stroop task. Whilst it is hard to disentangle the relative contributions of poor initial timing template, too-fast accumulation of pulses (leading to estimates that more time has passed than has) and response inhibition and working memory capacity to this pattern of performance it therefore seems likely that the overall pattern of results in the bvFTD group, tapping too fast and speeding up over time, is due at least in part to a fundamental deficit in timing, not just poor executive skills or working memory. Of note, a proportion of participants in each group did not show speeding up and more research is needed to investigate why that might be.

There was no evidence that grey matter volume or white matter structure in the ‘timing’ tracts were associated with performance on the externally-paced task; but this is not surprising given the relatively small deficits seen in this task, and the fact that the presence of a consistent external beat means the nature of this task is as much about reacting to the stimulus as it is about temporal prediction and timing *per se*. In contrast, faster mean IRI on the self-paced task, which was a deficit seen only in the bvFTD group, was associated with reduced grey matter volume in the cerebellum and right middle temporal gyrus, increased axial diffusivity in the right superior longitudinal fasciculus (a structure crucial in connecting frontotemporal and frontoparietal regions) and the suggestion of decreased fractional anisotropy in the left inferior cerebellar peduncle. The DTI findings support the notion that disconnections in white matter tracts between timing regions may lead to task-specific impairments (Ghajar and Ivry, 2008) and extend the literature implicating white matter tract integrity in finger-tapping ability in healthy individuals (Schulz et al., 2014; Ullén et al., 2008). Furthermore, together these neuroimaging findings are consistent with current theories of a cognitive timing network underlying explicit motor output within areas that have previously been identified as crucial to cognitive timing ability (e.g., Coull et al., 2013).

The role of the cerebellum in timing has been the subject of much debate, and in general reviews tend to suggest that it is crucial for sub-second timing, whilst its role in supra-second time is more debatable and more likely to vary depending on the nature of the timing task. In the bvFTD group presented here there were robust associations between this region and mean self-paced IRI with the target IRI of 1500 ms suggesting that reduced cerebellar volume contributed to fast tapping, and this would fit with the proposed role of the cerebellum in establishing a representation of a temporal stimulus. It has been suggested that the sub-supra-second cut-off for cerebellar involvement is too precise, and that the cerebellum does contribute to supra-second timing albeit with regions such as frontal cortices playing an increasing role as times get progressively longer (see e.g., Allman and Meck, 2012) and certainly our results would support this. The cerebellum is not commonly conceived as a locus of pathology within bvFTD. However extensive investigation into the neuropathological phenotype of those with a mutation of the newly discovered C9ORF72 gene responsible for many cases of bvFTD has identified the cerebellum as the greatest locus of pathological deposition and atrophy within those carrying this mutation (DeJesus-Hernandez et al., 2011; Mahoney, Beck, et al., 2012), and the current results suggest that the amount and role of cerebellar atrophy across the clinical spectrum of bvFTD merits further investigation.

In contrast to this finding there was little evidence of a relationship between cortical areas and performance on this task and no evidence of the involvement of those cortical areas thought to be particularly important for timing (for example the basal ganglia, SMA or frontal regions). This may be because there was relatively little atrophy in these regions so effects were small, as well as the small number in the analyses, but of note there was no evidence that executive function (Stroop) was associated with self-paced tapping in this cohort, and only evidence that working memory was associated with slower mean IRI across both patients and controls, so it may also be that the key pathology underlying the deficit seen here is cerebellar. The right middle temporal gyrus has also been associated with timing tasks, although more commonly with auditory attention for perceptual timing (Coull et al., 2004; Wiener et al., 2010) so further research is needed to elucidate its potential role in the deficits seen here.

These results have important clinical implications. The observation that some aspects of explicit motor timing are damaged in bvFTD, and that this maps onto neural correlates that have been consistently identified in both neurologically-compromised (Bechtel et al., 2010; O׳Boyle et al., 1996; Picton et al., 2007; Rowe et al., 2010) and healthy control (Coull et al., 2013; Ortuño et al., 2011) populations, provides some support for the existence of a network that supports cognitive timing ability. Parts of this network appear to be damaged in bvFTD and associated with impaired motor timing. Dysfunction in this and other cognitive timing skills may contribute to a number of phenotypic features particular to bvFTD. Dysfunctional social cognition is a hallmark behavioural feature of bvFTD recognised in diagnostic criteria (Rascovsky et al., 2011), and cognitive timing has been highlighted as an important contributing factor to social cognitive processes, such as in theory of mind (Baron-Cohen et al., 2001), or making sense of the temporal course of events (Allman et al., 2011; Grondin, 2010). More general investigation into how precise timing may be key to many different types of actions may also prompt developments in models of cognitive timing. For example, this study implicates the cerebellum as important for 1500m explicit timing. Had the paradigm somehow involved, for example, ‘comic timing’ in which a pause is required before delivering a punch line, we propose that that anatomical correlation with different hubs within the same timing network (e.g. cerebellum, basal ganglia and their inter-connections) plus other prefrontal regions more strongly implicated in social interaction, theory of mind and perspective taking might have been observed. Thus, the identification of timing deficits in bvFTD may develop our clinical understanding of the condition as subtle timing deficits may contribute to many of the more recognised social and behavioural features.

This study has several limitations that suggest directions for future work. Case numbers here were small, potentially limiting power to detect effects. This also prevented our examining whether the brain-behaviour relationship found in the bvFTD group was different to that in controls, and therefore although our findings are suggestive of a relationship that is unique to bvFTD and related to the associated atrophy, they are not conclusive of this. Future work should engage larger patient cohorts in order to address this. A key analysis of interest would also be to look separately at imaging correlates of participants exhibiting either negative (faster) or positive (slower) drift as we might predict that specific regions of the basal ganglia (“core timer”) or SMA (“accumulator”) would be associated with either speeding up or slowing down, but not both, which was all we had the power to examine here (using absolute drift). The results of this study should also be interpreted in the context of general cognitive dysfunction. Although in this cohort there was only evidence that the self-paced timing deficits were associated with working memory capacity (and in both patients and controls) and not with executive function (indexed by the Stroop), it would be informative to examine whether other aspects of executive function were related to tapping performance. Other dimensions of cognitive timing besides those investigated here, such as timing reproduction and estimation, and the relations between those dimensions and finger-tapping should also be explored. Longitudinal studies will be required to establish whether dysfunctional cognitive timing mechanisms may have any utility as a potential biomarker for bvFTD. Genetic mutations account for up to 40% of all cases of FTD (Rohrer et al., 2010), and thus identifying novel non-invasive and low-cost metrics of early detection of disease manifestation is of great importance, and therefore it would also be of interest to examine similar tasks in at-risk populations. Within the highly heterogeneous framework of phenotypic expression and pathological bases present in bvFTD, such a metric may also prove of use in differentiating between responsible pathological subtypes or pathogenic mutations.

In conclusion, the present study presents insights into the disruption of explicit motor timing in bvFTD in the context of intact performance in several other neurologically-compromised disease controls and healthy individuals. This deficit was associated with reduced cerebellar volume, and increased axial diffusivity in white-matter tracts known to connect subcortical and cortical ‘timing’ regions. These data are consistent with the hypothesis that deficits in cognitive timing may contribute to some of the behavioural abnormalities observed within bvFTD, and that the integrity of the white matter connecting previously implicated cortical and subcortical structures may be crucial in supporting intact timing ability.

## Figures and Tables

**Table 1:** Demographic and neuropsychological characteristics of participants.

	**Controls (*N*=31)**	**bvFTD (*N*=20)**	**svPPA (*N*=11)**	**naPPA (*N*=4)**	**AD (*N*=8)**
**Demographics**					
Age (years)	62.6 (7.2)	63.6 (9.6)	66.2 (7.3)	66.4 (13.1)	66.6 (5.9)
Gender (M:F)	14:17	17:3	7:4	2:2	7:1

**IQ**					
WASI vocab (/80)	70.7 (4.7)	43.6 (18.4)	**24.3 (17.3)**^**vs FTD**^	41.5 (5.9)	53.8 (12.8)
WASI blocks (/71)	46.1 (11.7)	**22.2 (14.9)**^**vs svPPA**^	32.7 (17.2)	25.8 (17.1)	20.9 (13.6)
WASI similarities (/48)	41.0 (6.2)	23.2 (10.4)	**13.5 (11.0)**^**vs FTD**^	23.5 (10.3)	26.9 (9.2)
WASI matrices (/32)	27.3 (8.3)	14.6 (6.3)	19.0 (8.7)	14.3 (6.6)	14.5 (8.2)
NART total (/50)^a^	41.8 (5.7)	30.1 (12.0)	23.7 (17.8)	19.3 (9.5)	33.4 (9.6)

**Episodic memory**					
RMT Words (/50)^b^	48.1 (2.6)	**36.1 (7.4)**^**vs naPPA**^	32.0 (8.3)	46.8 (3.4)^⁎^	34.7 (8.7)
RMT Faces (/50)^c^	43.3 (4.3)	**34.0 (6.6)**^**vs naPPA, AD**^	32.9 (8.8)	40.3 (3.2)^⁎^	38.2 (4.7)

**Semantic processing**					
BPVS (/150)	147.6 (1.6)	**130.1 (19.8)**^**vs naPPA**^	**75.8 (51.4)**^**vs FTD**^	141.8 (7.7)^⁎^	131.6 (22.8)

**Executive function**					
D*–*KEFS Stroop colour (s)^d^	29.5 (4.9)	39.9 (15.1)	53.0 (26.0)	**75.3 (25.1)**^**vs FTD**^	50.1 (15.5)
D*–*KEFS Stroop word (s)^d^	21.2 (3.6)	26.6 (10.5)	30.9 (11.4)	**72.3 (25.1)**^**vs FTD**^	30.9 (7.5)
D*–*KEFS Stroop inhibition (s)^d^	54.3 (12.7)	93.1 (41.0)	109.3 (46.0)	125.0 (43.7)	**136.5 (48.6)**^**vs FTD**^

**Other skills**					
GNT (/30)	25.2 (3.3)	**11.5 (7.6)**^**vs naPPA, AD**^	**1.7 (3.1)**^**vs FTD**^	18.5 (8.4)^⁎^	18.0 (5.4)
Forwards digit span (/12)	9.1 (1.5)	7.5 (2.7)	7.8 (3.2)^⁎^	5.8 (2.8)	7.0 (2.4)
Backwards digit span (/12)	7.2 (2.0)	5.7 (2.3)	6.0 (3.0)^⁎^	4.5 (2.1)	**3.9 (1.8)**^**vs FTD**^
GDA Addition (/12)^e^	7.5 (2.5)	4.9 (3.5)	5.1 (4.4)	**2.5 (1.9)**^**vs FTD**^	3.6 (1.5)
GDA Subtraction (/12)^e^	8.0 (2.6)	5.3 (3.9)	4.6 (4.7)	**2.0 (1.4)**^**vs FTD**^	4.1 (2.6)
VOSP Object decision (/20)	18.6 (1.6)	**15.5 (4.2)**^**vs naPPA**^	15.3 (4.2)	17.8 (2.6)^⁎^	16.9 (1.8)

Data are mean (SD) with the exception of gender. In general all patient groups performed, on average, worse than controls at all tests. Exceptions have the superscript ⁎ which indicates that there was no difference between that patient group and controls. Results in bold indicate that, on average, that patient group performed worse than another patient group on that test (indicated by the superscript). AD, Alzheimer׳s disease; BPVS, British picture vocabulary scale; bvFTD, behavioural variant frontotemporal dementia; D–KEFS, Delis–Kaplan Executive Function System; GDA, Graded Difficulty Arithmetic; GNT, Graded Naming Test; NART, National Adult Reading Test; naPPA, nonfluent/agrammatic progressive aphasia; RMT, recognition memory test; svPPA, semantic variant primary progressive aphasia; VOSP, Visual Object and Space Perception Battery; WASI, Wechsler Abbreviated Scale of Intelligence.

a 1 bvFTD, 2 svPPA and 1 naPPA unable to attempt the NART.

b 2 bvFTD, 1 svPPA and 1 AD unable to complete RMW.

c 1 bvFTD unable to complete RMF.

d 3 bvFTD and 1 svPPA unable to complete Stroop.

e 1 bvFTD and 1 AD unable to complete GDA.

**Table 2:** Externally-paced tapping: timing metrics for each group, from ARMAX model.

		**Control (*N*=31)**	**bvFTD (*N*=20)**	**svPPA (*N*=11)**	**naPPA (*N*=4)**	**AD (*N*=8)**
**Autocorrelation model statistics**						
Intercept (ms)^a^	Mean (SD)	1474 (37.8)	1480 (40.7)	1483 (15.0)	1507 (58.9)	1490 (53.2)
	Min, max	1396, 1583	1384, 1570	1452, 1500	1447, 1567	1428, 1605
Drift (ms)^b^	*N* (%) negative drift	9 (29)	7 (35)	4 (36)	2 (50)	2 (25)
	Mean (SD) drift	19 (32.3)	10 (98.1)	5 (25.4)	−86 (161.9)	6 (40.4)
	Mean (SD) absolute drift	29 (23.4)	63 (74.5)	19 (16.1)	109 (142.3)	30 (25.2)
	Mean (SD) negative drift	−17 (11.7)	−75 (106.5)	−20 (19.1)	−195 (175.7)	−49 (49.4)
	Mean (SD) positive drift	34 (25.5)	56 (54.6)	19 (15.7)	23 (9.8)	24 (14.6)
Modelled mean IRI (ms)	Mean (SD)	1494 (9.2)	1492 (36.3)	1497 (3.8)	1415 (157.3)	1497 (8.8)
	Min, max	1455, 1506	1389, 1604	1490, 1503	1179, 1500	1485, 1511
Lag 1 autocorrelation	*N* (%) between -0.5 and 0	24 (77)	15 (75)	6 (55)	2 (50)	8 (100)
	Mean (SD)	−0.33 (0.3)	−0.15 (0.20)	−0.45 (0.19)	−0.13 (0.44)	−0.24 (0.13)
	Min, max	−0.97, 0.34	−0.51, 0.21	−0.71, −0.07	−0.59, 0.45	−0.46, −0.08

**Variances**						
Inter-response interval variance (ms^2^)	Mean (SD)	11004 (9749)	18,573 (20,812)	5612 (6150)	35,232 (22,763)	9628 (6042)
	Min, max	600, 41,033	1566, 73,334	1290, 22,921	7946, 56,864	2393, 18,054
Clock variance (ms^2^)	Mean (SD)	3403 (10,605)	16,403 (26,946)	−212 (2338)	27,710 (47,858)	5256 (3937)
	Min, max	−30,825, 40,306	−160, 104,232	−5623, 2830	−10,098, 96,870	406, 11,654
Motor variance (ms^2^)	Mean (SD)	3800 (6114)	1085 (5653)	2912 (4047)	3761 (23,130)	2186 (1716)
	Min, max	−2470, 31,653	−15,449, 12,918	236, 14,272	−23,022, 33,481	696, 4777

Abbreviations: SD, standard deviation; bvFTD, behavioural variant frontotemporal dementia; naPPA, nonfluent/agrammatic progressive aphasia; svPPA, semantic variant primary progressive aphasia; AD, Alzheimer׳s disease.

a Note that the intercept for each participant is the first IRI for each participant, derived after having fitted the model across all taps for each participant. Thus the group means presented here are an indication of how well participants in each group, on average, judged the first tap.

b Drift is the difference between first and last IRI.

**Table 3:** Externally-paced tapping: mean (95% CI) difference between controls and each patient group, with and without adjustment for age and gender.

	**Estimate (95% CI)**	**bvFTD (*N*=20)**	**svPPA (*N*=11)**	**naPPA (*N*=4)**	**AD (*N*=8)**
Clock variance (ms^2^)	Unadjusted	**13,000 (3974, 31,331)**	−**3615 (**−**8273,** −**48)**	24,307 (−6768, 95,512)	1853 (−3253, 6341)
	Adjusted	**13,039 (1925, 30,692)**	−2723 (−9133, 3382)	25,349 (−8713, 99,028)	2717 (−5721, 9926)
Motor variance (ms^2^)	Unadjusted	−2716 (−6794, 31)	−888 (−3942, 2375)	−40 (−18,838, 29,831)	−1614 (−4699, 382)
	Adjusted	−2500 (−7260, 1569)	−1138 (−4942, 2429)	−428 (−21,022, 28,325)	−1714 (−6136, 1449)
Mean IRI (ms)	Unadjusted	−2 (−16, 18)	4 (0, 8)	−78 (−238, 3)	3 (−3, 11)
	Adjusted	0 (−21, 21)	6 (0, 19)	−77 (−237, 12)	6 (−3, 21)
Drift (ms)	Unadjusted	−9 (−66, 28)	−14 (−35, 3)	−105 (−343, 4)	−13 (−57, 8)
	Adjusted	−15 (−86, 29)	−18 (−43, 5)	−106 (−364, 6)	−21 (−69, 11)
Absolute drift (ms)	Unadjusted	**34** (**10, 82)**	−10 (−22, 3)	80 (−4, 294)	1 (−13, 26)
	Adjusted	**38** (**5, 99)**	−7 (−24, 10)	82 (−6, 309)	6 (−16, 36)

A positive effect is indicative of greater variance (clock and motor variance) or slower time (IRI and drift metrics) relative to controls. Effects in bold indicate statistically significant mean differences between the patient group and controls, *p*<0.05. IRI=inter-response interval, i.e. mean speed of tapping across the whole task; drift=difference between first and last tap; absolute drift=the modulus of drift. Abbreviations: CI, confidence interval; bvFTD, behavioural variant frontotemporal dementia; naPPA, progressive non-fluent aphasia; svPPA, semantic variant primary progressive aphasia, AD, Alzheimer׳s disease.

**Table 4:** Self-paced tapping: timing metrics for each group, from ARMAX model.

		**Control (*N*=28)**	**bvFTD (*N*=19)**	**svPPA (*N*=9)**	**naPPA (*N*=4)**	**AD (*N*=8)**
**Autocorrelation model statistics**						
Intercept (ms)	Mean (SD)	1409 (149.5)	1321 (263.2)	1285 (174.8)	1270 (128.7)	1457 (235.6)
	Min, max	1139, 1782	821, 1677	990, 1472	1138, 1382	981, 1787
Drift (ms)	N (%) negative drift	18 (64)	13 (68)	6 (67)	2 (50)	6 (75)
	Mean (SD) drift	−28 (202.8)	−129 (376.5)	−68 (166.5)	13 (164.9)	−190 (421.1)
	Mean (SD) absolute drift	155 (130.9)	299 (255.2)	127 (122.1)	115 (98.6)	296 (344.2)
	Mean (SD) negative drift	−142 (92.2)	−312 (237.9)	−146 (135.1)	−102 (57.5)	−324 (400.6)
	Mean (SD) positive drift	177 (185.6)	269 (311.5)	88 (103.1)	128 (158.6)	212 (89.4)
Modelled mean IRI	Mean (SD)	1368 (217.1)	1223 (306.8)	1242 (223.8)	1331 (162.7)	1282 (284.5)
	Min, max	1025, 1802	662, 1830	956, 1513	1148, 1544	801, 1631
Lag 1 autocorrelation	Number between −0.5 and 0	15 (54)	5 (26)	3 (33)	2 (50)	5 (63)
	Mean (SD)	−0.03 (0.2)	0.14 (0.22)	0.06 (0.17)	0.02 (0.28)	−0.02 (0.25)
	Min, max	−0.45, 0.28	−0.38, 0.47	−0.29, 0.35	−0.35, 0.25	−0.30, 0.36

**Variances**						
Inter-response interval variance (ms^2^)	Mean (SD)	4557 (3233)	15,666 (26,074)	3186 (1505)	53,971 (94,270)	11,711 (14,209)
	Min, max	747, 13,129	1147, 95,830	1829, 5732	3527, 195,331	950, 36,104
Clock variance (ms^2^)	Mean (SD)	4345 (4067)	16,033 (24,993)	3838 (2518)	75,495 (140,429)	16,710 (25,883)
	Min, max	173, 18,795	1365, 99,334	784, 7735	2333, 286,108	870, 62,286
Motor variance (ms^2^)	Mean (SD)	106 (1132)	−183 (9762)	−326 (579)	−10,762 (23,134)	−2499 (5925)
	Min, max	−2833, 3616	−15,389, 36,118	−1342, 522	−45,389, 2783	−13,091, 2060

Abbreviations: SD, standard deviation; min, minimum value; max, maximum value; bvFTD, behavioural variant frontotemporal dementia; naPPA, nonfluent/agrammatic progressive aphasia; svPPA, semantic variant primary progressive aphasia; AD, Alzheimer׳s disease. All effect sizes are versus control.

**Table 5:** Self-paced tapping: mean (95% CI) difference between controls and each patient group, with and without adjustment for age and gender.

	**Estimate (95% CI)**	**bvFTD (*N*=19)**	**svPPA (*N*=9)**	**naPPA (*N*=4)**	**AD (*N*=8)**
Clock variance (ms^2^)	Unadjusted	**11,688 (3821, 29,119)**	−506 (−2954, 1608)	71,150 (−782, 282,794)	12,365 (−1519, 39,648)
	Adjusted	8307 (−11,237, 28,371)	−2274 (−29,423, 2917)	70,585 (−1836, 288,526)	9093 (−9676, 33,592)
Motor variance (ms^2^)	Unadjusted	−290 (−3122, 6541)	−433 (−1041, 104)	−10,868 (−45,649, 1588)	−2606 (−8722, 558)
	Adjusted	226 (−3265, 6978)	−353 (−1720, 2782)	−11,002 (−46,602, 1640)	−2260 (−8029, 1365)
Total variance (ms^2^)	Unadjusted	**11,109** (**2298, 28,001)**	−1372 (−2972, 82)	**49,414** (**968, 191,580)**	7154 (−579, 21,413)
	Adjusted	8758 (−5382, 26,617)	−2979 (−20,101, 706)	**48,580** (**292, 195,303)**	4573 (−7900, 17,816)
Accuracy (ms)	Unadjusted	−145 (−305, 7)	−126 (−297, 35)	−37 (−199, 172)	−86 (−335, 106)
	Adjusted	**−175** (**−360, −26)**	−130 (−305, 40)	−28 (−192, 374)	−106 (−389, 76)
Continual drift (across time, ms)	Unadjusted	−101 (−277, 95)	−40 (−183, 83)	41 (−109, 276)	−163 (−586, 62)
	Adjusted	−77 (−268, 175)	−4 (−155, 149)	72 (−137, 426)	−120 (−520, 102)
Drift (absolute value, ms)	Unadjusted	**144** (**29, 282)**	−28 (−105, 95)	−39 (−138, 83)	142 (−17, 532)
	Adjusted	**168** (**38, 322)**	−21 (−118, 106)	−42 (−140, 69)	**160** (**14, 580)**

A positive effect is indicative of greater variance (clock and motor variance) or slower time (accuracy and drift metrics) relative to controls. Effects in bold indicate statistically significant mean differences between the patient group and controls, *p*<0.05. Abbreviations: CI, confidence interval; bvFTD, behavioural variant frontotemporal dementia; naPPA, progressive non-fluent aphasia; svPPA, semantic variant primary progressive aphasia, AD, Alzheimer׳s disease.

**Table 6:** Summary of VBM findings in the bvFTD group.

**Behavioural measure**	**Association**	**Peak coordinates**	**Brain regions**	**Cluster size (voxels)**	***t* Value**	***p* Value**
Self-paced mean IRI	Faster mean IRI associated with reduced GM volume	−10 −73 −47	left (peak) and right cerebellum	64,717	6.39	0.004
		55 −47 −5	right middle temporal gyrus	578	6.33	0.032

Findings are corrected for multiple comparison across the whole brain using family-wise error correction thresholded at *p*<0.05. Coordinates (mm) of peak *p* values are shown in Montreal Neurological Institute standard stereotactic space. Voxels were 1 mm^3^.
